# Preclinical evaluation of PAC1 targeting with radiolabeled Maxadilan

**DOI:** 10.1038/s41598-017-01852-8

**Published:** 2017-05-11

**Authors:** Lieke Joosten, Maarten Brom, Martin K. H. Schäfer, Otto C. Boerman, Eberhard Weihe, Martin Gotthardt

**Affiliations:** 1 Department of Radiology and Nuclear Medicine, Radboud university medical center, PO Box 9101, 6500 HB Nijmegen, The Netherlands; 2 Institute of Anatomy and Cell Biology, Dept. of Molecular Neuroscience, Philipps University of Marburg, Robert-Koch-Strasse 8, 35037 Marburg, Germany

## Abstract

There is an ongoing search for new tracers to optimize imaging of beta cell-derived tumors (insulinomas). The PAC1 receptor, expressed by insulinomas, can be used for targeting of these tumors. Here, we investigated whether radiolabeled maxadilan could be used for insulinoma imaging. Maxadilan was C- or N-terminally conjugated with DTPA (termed maxadilan-DPTA or DTPA-maxadilan respectively). BALB/c nude mice bearing subcutaneous INS-1 tumors were injected with either In-111-labeled maxadilan-DTPA or In-111-DTPA-maxadilan. Biodistribution studies were carried out at 1, 2 and 4 hours after injection and SPECT/CT imaging 1 and 4 hours after injection of maxadilan-DTPA-^111^In. Radiolabeling of maxadilan-DTPA (680 MBq/nmol) was more efficient than of DTPA-maxadilan (55 MBq/nmol). Conjugation with DTPA slightly reduced receptor binding affinity *in vitro*: IC_50_ values were 3.2, 21.0 and 21.0 nM for maxadilan, ^nat^In-DTPA-maxadilan and maxadilan-DTPA-^nat^In respectively. Upon i.v. injection maxadilan-DTPA-^111^In accumulated specifically in INS-1 tumors (7.30 ± 1.87%ID/g) and in the pancreas (3.82 ± 0.22%ID/g). INS-1 tumors were clearly visualized by small animal SPECT/CT. In conclusion, this study showed that the high affinity of maxadilan to the PAC1 receptor was maintained after DTPA conjugation. Furthermore, radiolabeled maxadilan-DTPA accumulated specifically in INS-1 tumors and, therefore, may qualify as a useful tracer to image insulinomas.

## Introduction

The most common pancreatic neuroendocrine tumors (NETs) are insulinomas. Insulinomas are tumors derived from the insulin-producing beta cells located in the islets of Langerhans in the pancreas. Clinical symptoms include symptomatic hypoglycemias as well as weight gain (due to increased food intake to compensate for hypoglycemia) and neurological symptoms^1^. Biochemical diagnosis is based on low blood glucose levels or elevated proinsulin, insulin and C-peptide levels in plasma and these tests can be complemented with fasting tests (which result in hypoglycemia if insulinoma is present)^1–3^. The most widely used nuclear medicine imaging strategy for detecting insulinomas is somatostatin receptor scintigraphy (SRS) using ^111^In-labeled octreotide. SRS is based on targeting somatostatin receptors (SSTR), which are over-expressed in sixty percent of insulinomas^4^. Because the remaining forty percent has either low or no expression of this receptor, many insulinomas are not being detected by scintigraphy based on somatostatin receptor targeting^2–4^. More preferably, PET (Positron Emission Tomography) imaging with octreotide analogues can be used, since PET is more sensitive than SPECT imaging, due to the higher spatial resolution of clinical PET scanners and higher sensitivity in detection of emitted gamma photons. A few promising studies were conducted using SSTR PET/CT, although these studies showed that a small percentage of insulinomas remain undetected^5–8^.

More than ten years ago, it was demonstrated that almost all insulinomas express several neuropeptide receptors, including CCK_2_ (cholecystokinin), VPAC1 (vasoactive intestinal peptide/PACAP receptor subtype 1) and GLP-1 (glucagon-like peptide 1) receptors^4^. Several preclinical SPECT and PET studies in RipTag2 mouse models using the GLP-1 analogues exendin-3 and exendin-4, showed specific tracer accumulation in insulinomas via the GLP-1 receptor^9–11^. In the first pioneering clinical PET/CT and SPECT/CT studies with exendin-4 in insulinoma patients, small insulinomas, which could not be detected by conventional imaging methods (endoscopic ultrasound, MRI (Magnetic Resonance Imaging), CT (Computed Tomography), SRS)^12–17^, were accurately diagnosed. The success of visualizing neuroendocrine tumors is partially dependent on the type of receptors expressed in the tumors, as was shown previously by Baumann *et al*.^5^. For example, malignant insulinomas have a low incidence and expression density of the GLP-1 receptor, but show high expression of the somatostatin receptor, where in benign insulinomas the expression profile is more favorable for the GLP-1R^4, 18, 19^. Therefore, to improve the diagnostic tool box, the search for new tracers for detection of insulinomas is ongoing.

Vasoactive intestinal peptide (VIP)/pituitary adenylate cyclase activating polypeptide (PACAP), and their receptors PAC1, VPAC1 and PAC2 have emerged as important factors in islet cell function (insulin secretion) and growth and differentiation of neuroendocrine tumors including insulinomas. As these receptors are highly expressed in insulinomas and beta cells, they are potential targets for beta cell and insulinoma imaging^20–22^.

Using *in vitro* receptor autoradiography with VPAC1 and VPAC2 subtype-selective ligands, respectively, insulinomas have been demonstrated to bind high levels of radiolabeled selective VPAC1 receptor ligands, although substantially less than GLP1 selective ligands^4^. As PAC1, VPAC1 and VPAC2 mRNAs were detected in a rat insulinoma cell line with PAC1 binding prevailing over that of VPAC1 and VPAC2^23^, the PAC1 receptor seemed to be a promising alternative for targeting insulinomas. The natural mammalian ligands at the PAC1 receptor are PACAP38 and the truncated form PACAP27. They bind to PAC1, VPAC1 and VPAC2, whereas VIP only binds to VPAC1 and VPAC2. Since PACAP38 and PACAP27 are non-selective PAC1/VPAC1/VPAC2 ligands and unstable in plasma^24^, they are not suited for *in vivo* targeting of PAC1 receptors. More than two decades ago it was demonstrated that the stable analogue maxadilan shared features with ligands of the PACAP family^25, 26^. Maxadilan is a 61-amino acid vasoactive peptide, derived from sand flies^27, 28^, and has been demonstrated to bind specifically and with high affinity to the PAC1 receptor but not to either VPAC1 or VPAC2^28^.

In the present study we have examined the ability of ^111^In-labeled maxadilan to image rat insulinoma xenografts (INS-1) in a nude mouse model. For this purpose maxadilan was either C- or N-terminally conjugated with DTPA to allow labeling with ^111^In. These compounds were evaluated for their radiolabeling properties and *in vitro* and *in vivo* binding characteristics.

## Results

### Radiolabeling

^111^In-DTPA-maxadilan and maxadilan-DTPA-^111^In could be labeled with a specific activity of 55 and 680 MBq/nmol, respectively. The radiochemical purity after purification exceeded 95%.

### Serum stability

**Figure 1:**
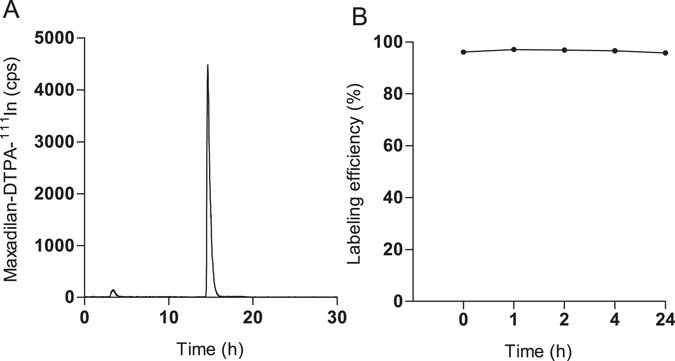
Stability of maxadilan-DTPA-^111^In in human serum. (**A**) HPLC profile of maxadilan-DTPA-^111^In directly after labeling. (**B**) Stability analysis of maxadilan-DTPA-^111^In before and 1, 2, 4 and 24 h after incubation in human serum at 37 °C.

The stability of maxadilan-DTPA-^111^In was analyzed in human serum. The radiochemical purity directly after labeling was >95% (Fig. 1A). The radiolabeled peptide remained intact up to 24 hrs after incubation in human serum, as is shown in Fig. 1B. ^111^In-EDTA and radiolabeled maxadilan have a retention time of 3–4 min and 14.6 min, respectively.

### Competitive binding assay

**Figure 2:**
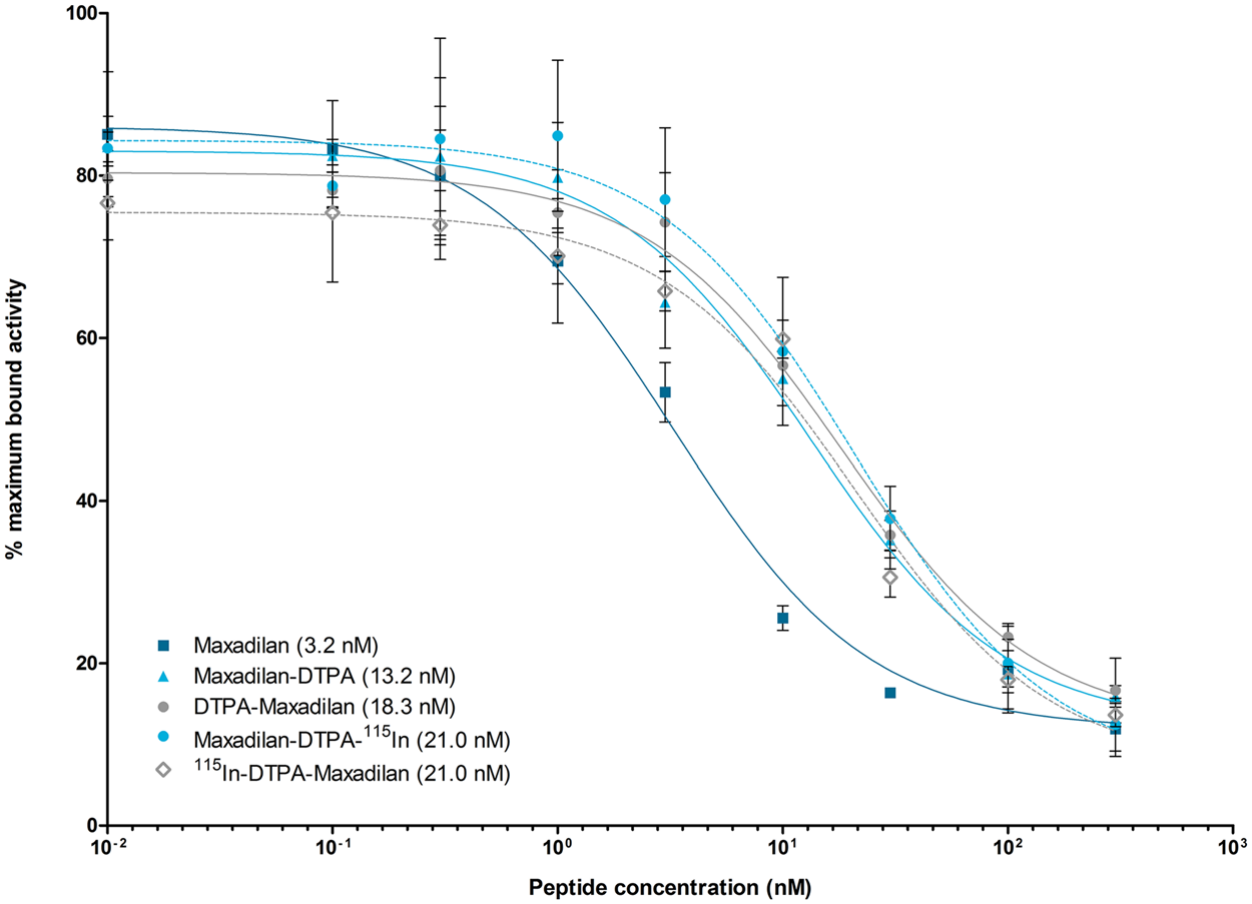
Competitive binding assay (IC_50_) on INS-1 cells of unlabeled maxadilan, maxadilan-DTPA and DTPA-maxadilan, either unlabeled, or labeled with ^115^In. ^111^In-labeled maxadilan-DTPA was used as radioligand. The table shows the IC_50_ values in nM.

The results of the IC_50_ determination of labeled and unlabeled maxadilan analogues are summarized in Fig. 2 and Table 1. Unlabeled maxadilan had the highest affinity (IC_50_ value: 3.2 nM) for the receptor. DTPA conjugation resulted in a somewhat lower affinity as indicated by slightly higher IC_50_ values: 18.3 and 13.2 nM (p < 0.001 and p < 0.001) for DTPA-maxadilan and maxadilan-DTPA, respectively. Labeling of DTPA-maxadilan or maxadilan-DTPA with ^115^In did not significantly reduce the affinity (p = 0.07 and p = 0.12 respectively) compared to the unlabeled compounds.

**Table 1:** IC_50_ values and 95% confidence interval (in nM) of the competitive binding assay on INS-1 cells.

Peptide	IC_50_ (nM)	95% confidence interval (nM)
Maxadilan	3.2	2.1–4.9
Maxadilan-DTPA	13.2	8.8–19.8
DTPA-Maxadilan	18.3	13.2–25.3
Maxadilan-DTPA- ^Nat^In	21.0	13.7–32.2
^Nat^In-DTPA-Maxadilan	21.0	11.8–37.3

### Biodistribution studies

**Figure 3:**
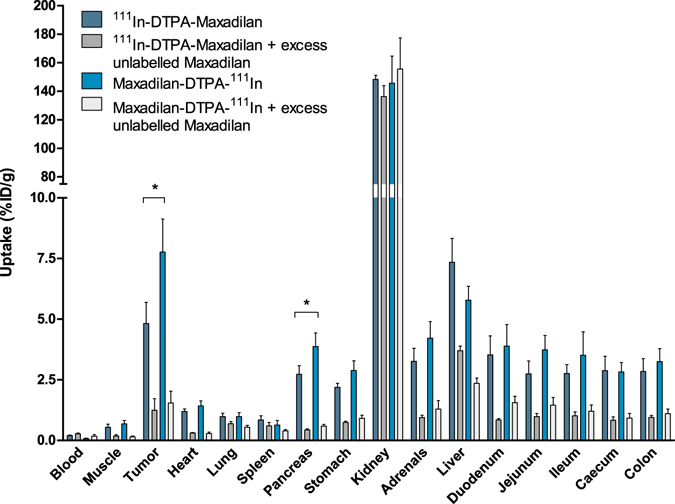
Biodistribution of ^111^In-labeled DTPA-maxadilan and maxadilan-DTPA in BALB/c nude mice bearing subcutaneous INS-1 tumors. Values are expressed as a percentage of the injected dose per gram of tissue (n = 5 mice per group, error bars SD). Blocking was performed by coinjection of a 100-fold excess of unlabeled maxadilan. Mice were dissected 2 hours after injection.

**Figure 4:**
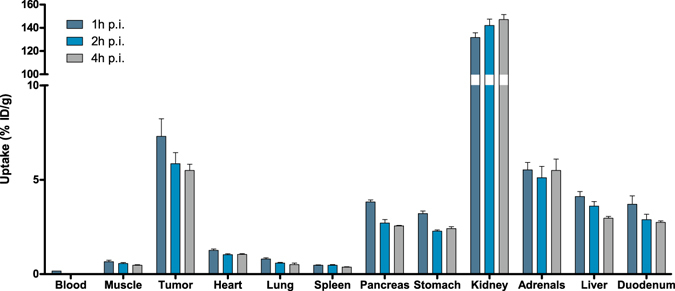
Biodistribution of ^111^In-labeled maxadilan-DTPA in BALB/c nude mice bearing subcutaneous INS-1 tumors. Values are expressed as a percentage of the injected dose per gram of tissue (n = 4 mice per group, error bars SD). Mice were dissected 1, 2 and 4 hours after injection to study the pharmacokinetics.

The results of the *in vivo* PAC1 receptor targeting study with ^111^In-labeled maxadilan in BALB/c nude mice bearing subcutaneous INS-1 tumors are summarized in Fig. 3. Uptake of maxadilan-DTPA-^111^In in the tumor was 7.76 ± 1.37%ID/g, which was significantly higher than that of ^111^In-DTPA-maxadilan (4.82 ± 0.87%ID/g) (p = 0.0037). Also, pancreatic uptake was significantly higher for maxadilan-DTPA-^111^In compared to ^111^In-DTPA-maxadilan, (3.87 ± 0.56%ID/g and 2.73 ± 0.35%ID/g respectively, p = 0.0048). Accumulation in both tumor and pancreas could be blocked by an excess of unlabeled maxadilan, demonstrating specific uptake of the peptides via the PAC1 receptor (1.54 ± 0.49%ID/g and 1.24 ± 0.48%ID/g for maxadilan-DTPA-^111^In and ^111^In-DTPA-maxadilan respectively in tumor tissue and 0.59 ± 0.07%ID/g and 0.43 ± 0.04%ID/g respectively for pancreatic uptake). Renal uptake was very high and similar for both peptides (148 ± 3 and 145 ± 19%ID/g for ^111^In-DTPA-maxadilan and maxadilan-DTPA-^111^In, respectively), which could not be blocked with an excess of unlabeled maxadilan. Furthermore, in all other dissected organs, except for blood and spleen, receptor-mediated uptake of maxadilan was observed. Since these results showed that maxadilan-DTPA-^111^In has higher accumulation in tumor and pancreatic tissue than ^111^In-DTPA-maxadilan, this peptide was used to study the pharmacokinetics. Figure 4 shows the fast clearance of the peptide from the blood. Furthermore, tumor accumulation peaked at one hour after injection (7.30 ± 1.87%ID/g) and decreased slightly over time (5.85 ± 1.17%ID/g and 5.49 ± 0.66%ID/g after 2 and 4 hours (not significant)). A similar trend was observed for the uptake in the pancreas: 3.82 ± 0.22%ID/g at 1 h p.i., 2.71 ± 0.36%ID/g at 2 h p.i. and 2.55 ± 0.07%ID/g at 4 h p.i.. Renal uptake increased over time, while the specific accumulation in the liver decreased. Table 2 gives an overview of the tumor-to-normal-organ ratios for ^111^In-labeled maxadilan-DTPA.

**Table 2:** Tumor-to-normal-organ ratios for ^111^In-labeled maxadilan-DTPA. Mean ± SD are shown.

Time p.i. (h)	Tumor-to-Blood	Tumor-to-Muscle	Tumor-to-Pancreas	Tumor-to-Kidney
1	44.62 ± 10.63	11.45 ± 3.75	2.01 ± 0.35	0.06 ± 0.02
2	139.65 ± 24.28	10.59 ± 2.25	2.34 ± 0.35	0.04 ± 0.01
4	314.74 ± 40.82	11.83 ± 1.91	2.04 ± 0.23	0.04 ± 0.01

### SPECT of BALB/c nude mice bearing subcutaneous INS-1 tumors

**Figure 5:**
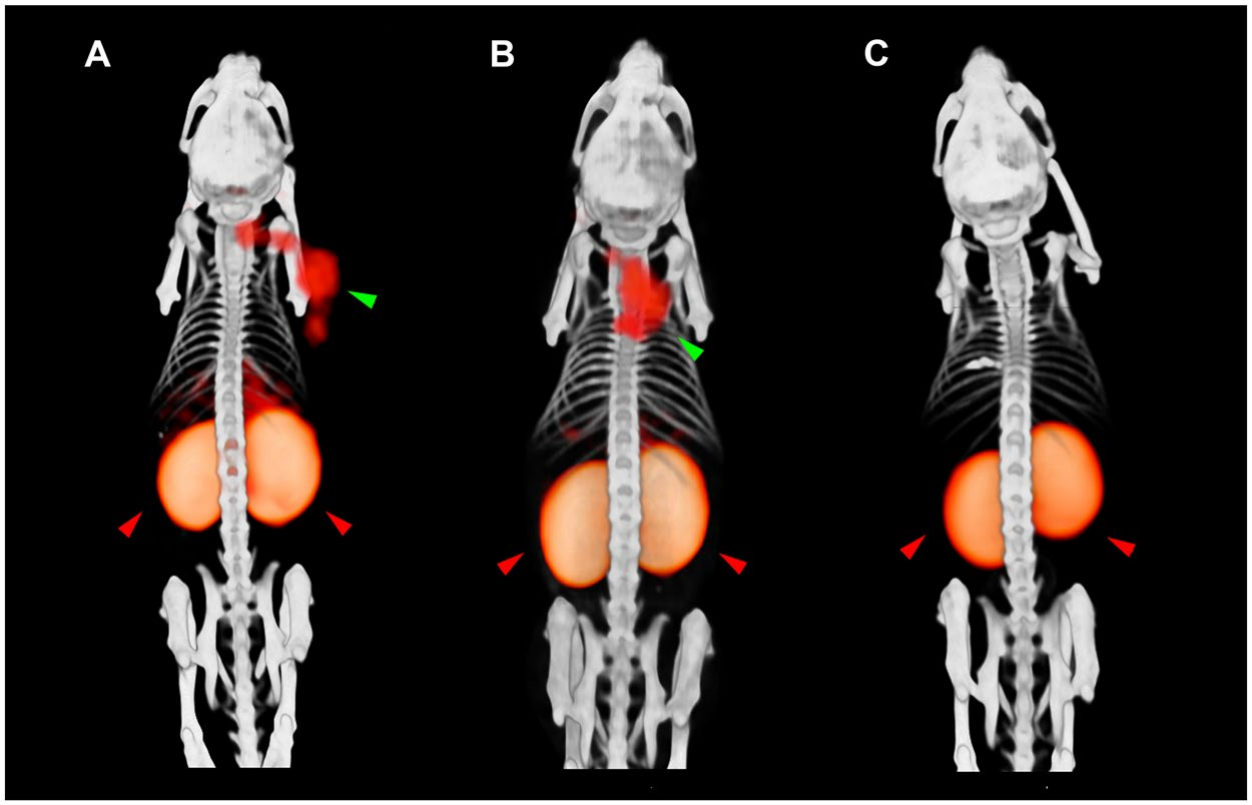
Fused SPECT/CT images of BALB/c nude mice bearing subcutaneous INS-1 tumors on the right shoulder (green arrow), obtained one and four hours p.i.. Image of a mouse injected with 9 MBq of maxadilan-DTPA-^111^In, 1 h (**A**), 4 h (**B**), and a mouse with 9 MBq of maxadilan-DTPA-^111^In and a 100 fold excess of unlabeled maxadilan (**C**). Kidneys are indicated with red arrows. Uptake in the abdomen is also seen, mainly in liver (**A** and **B**).

SPECT/CT images of BALB/c nude mice bearing subcutaneous INS-1 tumors are shown in Fig. 5.

Images were acquired one and four hours after injection of ^111^In-labeled maxadilan-DTPA and INS-1 tumors in the right shoulder were clearly visualized. In addition to tumor and kidney, accumulation of the tracer in the liver was observed. Figure 5C shows the image of a mouse which was co-injected with an excess of unlabeled maxadilan. The tumor on the right shoulder was not visualized with SPECT/CT, demonstrating specific uptake via the PAC1 receptor.

## Discussion

We demonstrate here for the first time that indeed radiolabeled maxadilan is a promising new tool to image subcutaneously transplanted insulinomas in mice.

We were able to radiolabel maxadilan with ^111^In with high specific activity while preserving the affinity for the PAC1 receptor. High and specific uptake of this tracer in both subcutaneous INS-1 tumors and pancreas was found. SPECT imaging clearly visualized the s.c. INS-1 xenografts. Both, pancreatic as well as tumor uptake were receptor mediated, as was demonstrated by blocking experiments with unlabeled maxadilan.

Since the conjugation of a DTPA moiety can affect the affinity of the peptide for the receptor, the peptide was conjugated with DPTA at either the C- or the N-terminus and the molecules were compared. The maximum specific activity of maxadilan-DTPA-^111^In was at least twelve times higher than that of ^111^In-DTPA-maxadilan. This could possibly be explained by hindrance of the radiometal incorporation into the DTPA molecule, due to either the secondary or tertiary structure of maxadilan or a conformational change in the peptide as a result of the conjugation.

Conjugation of DTPA to either the C-or N-terminus of maxadilan influenced the receptor binding affinity significantly. Maxadilan contains four cysteine residues, which form two disulfide bridges, one of which is located close to the N-terminus. Furthermore, it is known that the N-terminal residues of maxadilan are important for receptor binding^23, 29^. Therefore, it was thought likely that conjugation of DTPA to the N-terminus could influence the binding affinity. However, it has also been shown in previous studies that when the disulfide bridge at the N-terminal part of maxadilan is removed or changed, the receptor binding capacity of maxadilan is preserved^29, 30^. Nevertheless, as was determined in our *in vivo* biodistribution studies, the C-terminally modified maxadilan-DTPA-^111^In analogue showed higher uptake in the tumor, therefore this compound was used for further characterization.

The biodistribution shows specific accumulation of maxadilan in most organs. This is in line with the widespread PAC1 receptor expression and function in the pituitary, adrenal medulla, pancreas, stomach, colon, lung, and heart^31, 32^. As demonstrated in the SPECT images, maxadilan-DTPA-^111^In clearly visualized the subcutaneous INS-1 tumors and they were easily distinguished from the specific accumulation of radiolabeled maxadilan in various other tissues.

Other preclinical studies using INS-1 tumour-bearing mice have shown that GLP-1 receptor targeting ligands, labeled either with ^111^In, ^68^Ga or ^18^F, show a more favorable biodistribution compared to this maxadilan tracer. Although those previously-studied tracers show high tumor and kidney uptake similar to maxadilan, the tumor-to-normal-organ ratios are more optimal, which can be correlated with expression levels of the different receptors that are targeted^9, 33, 34^. Future studies need to be conducted to compare radiolabeled maxadilan with existing relevant PET agents in animals, before moving to clinical trials.

Recently, clinical imaging techniques such as ^68^Ga-labeled somatostatin receptor imaging and radiolabeled GLP-1 receptor imaging were introduced for diagnosis of insulinomas. These tracers would be useful for differentiating benign from malignant tumors or in diagnosis of metastatic NETS. However, the sensitivity is not optimal and false positive or false negative cases continue to be described using somatostatin receptor targeting^8, 19, 35^. False negative-SSTR PET/CT could be explained by small lesions (missed because the resolution of the scanner is not sufficient to detect them) or low somatostatin receptor expression (leading to a low signal which cannot be detected as the sensitivity of the scanner is not sufficient). A solution to the latter problem would be the use of a combination of different radiotracers, thus targeting alternative receptors. This was also suggested by Reubi *et al*., who conducted an elegant *in vitro* study proving that a cocktail of three different ligands was able to detect all tested NETs^36^. Tumors with no or low receptor density, which would be missed *in vivo* when using only one radiotracer, have a higher probability of being detected when multiple receptors are targeted. In addition, if one of the tracers had a very high uptake in the tumor, this high uptake could help to overcome the limited spatial resolution and also visualization of small lesions.

In addition to diagnostic imaging, some somatostatin analogues, when radiolabeled with a beta emitter, are used as therapeutic agents^37^. Due to the high accumulation of radiolabeled maxadilan in the kidneys, and to a lesser extent in other organs, the feasibility of peptide receptor radionuclide therapy with maxadilan would be questionable. Insulinomas may not be the only NETs in which radiolabeled maxadilan could play a diagnostic role. In a study carried out by Pisegna *et al*. high expression levels of the PAC1 receptor in rat gastric ECL (enterochromaffin-like) cells were observed^38, 39^. Currently, ^111^In-DTPA-octreotide or ^68^Ga-DOTATOC/^68^Ga-DOTA-octreotide are used for the detection of gastric neuroendocrine tumors, which originate from ECL cells^40^. Furthermore, PAC1 receptor expression has been demonstrated in Lewis lung tumour transplants^41^. Radiolabeled maxadilan could therefore be a possible candidate for detecting both ECL derived tumors and a subtype of small cell lung cancer, in addition to somatostatin receptor tomography. Moreover, there is evidence that there is high expression of PAC1 in human tumors such as paragangliomas, neuroblastomas, pituitary adenomas and endometrial cancers^42–45^ with expression found also in human lung cancers^46^. Importantly, most of these studies were conducted *in vitro*, so *in vivo* studies are needed to explore the ratio between peptide uptake in PAC1 receptor positive tumors and that in normal tissues where PAC1 is widely expressed.

In conclusion, radiolabeled maxadilan accumulates efficiently and specifically in INS-1 tumors and could potentially be used for *in vivo* PAC1 targeting in patients with insulinoma. Radiolabeled maxadilan, therefore, represents a new tracer to image insulinomas using SPECT in addition to or in combination with octreotide and exendin for the identification of benign or malignant insulinoma. Furthermore, the expression of PAC1 in other human tumors indicates a broader application for the usage of radiolabeled maxadilan. Thus, the newly generated PAC1 selective high affinity radiopeptide maxadilan can be employed for multireceptor tumour targeting *in vivo*.

## Materials and Methods

### Peptides

DTPA-conjugated and native maxadilan were purchased from Think Peptides (ProImmune Limited, Oxford, United Kingdom). N-terminal conjugation of DTPA (DTPA-Maxadilan) was performed with isothiocyanate-DTPA (Macrocyclics, Dallas, TX, USA) via a beta-alanine spacer and C-terminal conjugation (Maxadilan-DTPA) with *p*-NH2-Bn-DTPA via an extra C-terminal glutamic acid. The quality and purity of the peptides was determined using RP-HPLC (reversed-phase high performance liquid chromatography) and mass spectrometry (MS) by the manufacturer. RP-HPLC showed a purity of 95.35%, 93.9% and 100% and a retention time of 15.18 min, 15.81 min and 14.46 for maxadilan, DTPA-maxadilan and maxadilan-DTPA respectively. With MS the expected mass of the peptides was confirmed (6870, 7537 and 7641 Da for maxadilan, DTPA-maxadilan and maxadilan-DTPA respectively). The amino acid sequences of the peptides are shown in Table 3

**Table 3:**
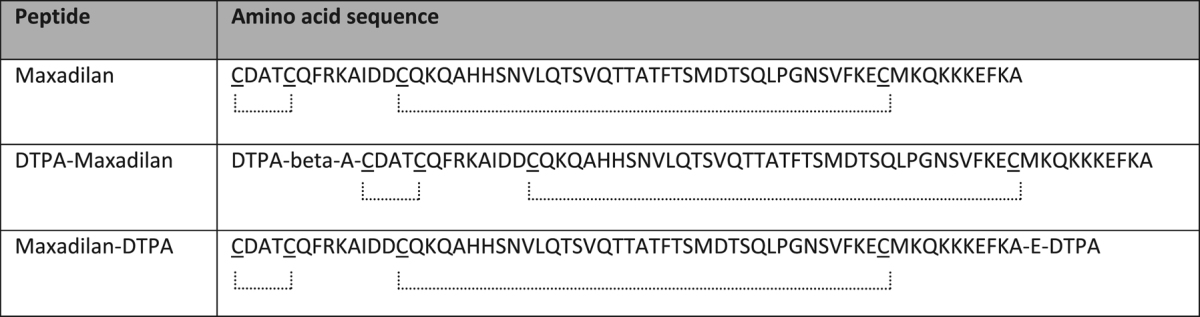
Amino acid sequences of maxadilan, DTPA-maxadilan and maxadilan-DTPA. Cysteine residues that form disulfide bonds are underlined and indicated by the dotted lines^30^.

.

### Radiolabeling

DTPA-maxadilan and maxadilan-DTPA were labeled with ^111^In by adding ^111^InCl_3_ to 1 µg peptide in 0.1 M MES buffer, pH 5.5. After incubation at RT for 20 min, 50 mM EDTA (ethylenediaminetetraacetic acid) (Sigma Aldrich, St. Louis, MO, USA) was added to a final concentration of 5 mM. Quality control was performed by Instant Thin Layer Chromatography (ITLC) on silica gel ITLC strips (Pall Corporation Life Sciences, New York, NY, USA). As a mobile phase 0.1 M EDTA in 0.1 M NH_4_Ac, pH 5.5 was used (R_f_^111^In-maxadilan = 0, R_f_^111^In-EDTA = 1). The reaction mixture was purified on a disposable PD-10 desalting column (GE Life Sciences, Diegem, Belgium), which was eluted with 10 ml PBS, 0.5% (v/w) bovine serum albumin (BSA) and fractions containing radiolabeled maxadilan were pooled.

### Serum stability

Maxadilan-DTPA was labeled with 5 MBq ^111^InCl_3_ as previously described and incubated with human serum (1:10) at 37 °C. Before addition of human serum and 1, 2, 4 and 24 hrs after incubation with human serum, samples were taken, mixed with acetonitrile (1:1) and centrifuged for 5 min at 5000 g to precipitate serum proteins. The supernatant was analyzed using RP-HPLC on a C_18_ reversed-phase column (Alltima; 4.6 mm × 25 cm; Grace, Breda, The Netherlands) and ITLC. The column was eluted with a linear gradient of 0.1% TFA (trifluoroacetic acid, Lab-Scan, Analytical Sciences, Brussels, Belgium) in acetonitrile (3% to 100% over 10 min) with a flow rate of 1 ml/min. ITLC was performed as described above.

### Cell culture

The rat insulinoma cell line INS-1^47^ was maintained in RPMI-1640 medium supplemented with 10% fetal bovine serum, 2 mM glutamine, 10 mM HEPES, 50 µM β-mercaptoethanol, 1 mM sodium pyruvate, 100 units/ml penicillin and 100 µg/ml streptomycin, in a humidified 5% CO_2_ atmosphere at 37 °C. The cells were harvested by trypsinization with trypsin/EDTA.

### IC_50_ determination

The 50% inhibitory concentration (IC_50_) of maxadilan, DTPA-maxadilan, maxadilan-DTPA, ^115^In-DTPA-maxadilan and maxadilan-DTPA-^115^In was determined using suspensions of INS-1 cells. Labeling of DTPA-maxadilan and maxadilan-DTPA with ^nat^In was performed as previously described^9, 48^. Unlabeled and ^115^In-labeled peptides were added to the cells (approximately 10 × 10^6^ cells in a final volume of 0.5 mL) in eppendorf tubes to final concentrations ranging from 0.1 to 300 nmol (n = 3) together with 1,000 Bq maxadilan-DTPA-^111^In. After 4 h incubation on ice, the cells were centrifuged at 3,000 × g, the supernatant was removed and the cells were washed with 1 ml ice-cold PBS and the radioactivity in the cell pellet was determined in a well-type gamma counter (Wallac 1480-Wizard, Perkin-Elmer, Boston, MA, USA). The IC_50_ value was calculated by one-site competition analysis with Graphpad Prism (version 5.03, GraphPad Software, San Diego, CA USA).

### Biodistribution studies

All experiments were performed in accordance with Radboud University guidelines. Animal experiments were approved by the Animal Ethical Committee of the Radboud University, Nijmegen, The Netherlands.

In order to assess the feasibility of targeting insulinomas with radiolabeled maxadilan, female BALB/c nude mice (6–8 weeks old) were injected subcutaneously with INS-1 cells (1 × 10^7^ cells in 200 µl). When the tumors had grown to approximately 5 mm in diameter, groups of five mice were injected with either 370 kBq ^111^In-DTPA-maxadilan or maxadilan-DTPA-^111^In (peptide dose: 13 pmol). For both peptides, an additional group of 5 mice was co-injected with an excess (1,300 pmol) of unlabelled maxadilan to determine the nonspecific binding of the peptides to the cells. Mice were euthanized 2 h p.i. and blood, muscle, tumor, heart, lung, spleen, pancreas, stomach, intestine, adrenals, kidney and liver were dissected, weighed and the radioactivity concentration was determined.

To examine the pharmacokinetics of maxadilan-DTPA-^111^In, BALB/c nude mice (n = 4/group) were injected with 370 kBq maxadilan-DTPA-^111^In and mice were euthanized at 1, 2 or 4 h p.i. The radioactivity concentration in the organs was measured in a gamma counter.

### SPECT of BALB/c nude mice bearing subcutaneous INS-1 tumors

BALB/c nude mice bearing subcutaneous INS-1 tumors were injected intravenously with 9 MBq maxadilan-DTPA-^111^In (13 pmol). A separate group of tumor-bearing mice was co-injected with an excess of unlabelled maxadilan (1,300 pmol). One and four h p.i. SPECT/CT images were acquired using a dedicated small animal SPECT scanner (U-SPECT-II, MILabs, Utrecht, The Netherlands). SPECT images were acquired with a 0.6 mm pinhole mouse collimator with an acquisition time of 50 min. The images were reconstructed with OSEM (3 iterations, 16 subsets, voxel size 0.375) using the U-SPECT-Rec software (MILabs, Utrecht, The Netherlands). The settings for the CT were as follows: spatial resolution, 160 μm; 40 kV; 612 μA.

### Statistical analysis

Statistical analysis was done using GraphPad Prism version 5.03 for Windows. The unpaired *t* test was used for determination of significance. A *p*-value below 0.05 was considered as significant. For the competitive binding assay the F-test was used to manually calculate significance.
